# A novel intrusion detection framework for optimizing IoT security

**DOI:** 10.1038/s41598-024-72049-z

**Published:** 2024-09-18

**Authors:** Abdul Qaddos, Muhammad Usman Yaseen, Ahmad Sami Al-Shamayleh, Muhammad Imran, Adnan Akhunzada, Salman Z. Alharthi

**Affiliations:** 1 Department of Computer Science, COMSATS University Islamabad (CUI), Islamabad, 45550 Pakistan; 2 Department of Data Science and Artificial Intelligence, Faculty of Information Technology, Al-Ahliyya Amman University, Amman, 19328 Jordan; 3 College of Computing and Information Technology, University of Doha for Science and Technology, Doha, 24449 Qatar; 4 Department of Software Engineering, College of Computing, Umm Al-Qura University, Mecca, 24381 Kingdom of Saudi Arabia

**Keywords:** Computer science, Information technology

## Abstract

The emerging expanding scope of the Internet of Things (IoT) necessitates robust intrusion detection systems (IDS) to mitigate security risks effectively. However, existing approaches often struggle with adaptability to emerging threats and fail to account for IoT-specific complexities. To address these challenges, this study proposes a novel approach by hybridizing convolutional neural network (CNN) and gated recurrent unit (GRU) architectures tailored for IoT intrusion detection. This hybrid model excels in capturing intricate features and learning relational aspects crucial in IoT security. Moreover, we integrate the feature-weighted synthetic minority oversampling technique (FW-SMOTE) to handle imbalanced datasets, which commonly afflict intrusion detection tasks. Validation using the IoTID20 dataset, designed to emulate IoT environments, yields exceptional results with 99.60% accuracy in attack detection, surpassing existing benchmarks. Additionally, evaluation on the network domain dataset, UNSW-NB15, demonstrates robust performance with 99.16% accuracy, highlighting the model’s applicability across diverse datasets. This innovative approach not only addresses current limitations in IoT intrusion detection but also establishes new benchmarks in terms of accuracy and adaptability. The findings underscore its potential as a versatile and effective solution for safeguarding IoT ecosystems against evolving security threats.

## Introduction

The Internet of Things (IoT) is an interconnection of smart devices when integrated with each other into a single network via different services or protocols. The IoT enables us to gather sensitive information from smart devices and to perform important operations, also allows smart devices to communicate with each other at high speed. Cloud services are used for maintaining backend processing and controlling information remotely in an IoT environment^1^. In an IoT network, different types of sensors are deployed to extract information, and then artificial intelligence (AI)-based algorithms are used to analyze the data for further actions. Web services and mobile applications are deployed in an IoT environment for front-end users to access information and connected devices^2^.

Many organizations are deploying IoT devices in operational technology and information technology environments to increase the efficiency and performance of the company. It is reported that 84 billion IoT devices will be operational by 2024 for many IoT applications such as smart electrical grid stations, intelligent transportation, smart homes, smart vehicular system, smart cities etc^3,4^. However, new cybersecurity risks have emerged with the rise of the IoT environment which become a threat to the security and safety of the operational ecosystem^5^. The newly emerged IoT threats are not only a risk for physical operations but also have financial impacts due to the failure of technology. Moreover, the IoT environment has brought multiple insecurities for social networks, businesses and critical IT infrastructure due to the threat of attack^6^. Therefore, the deployment of strategies for detecting threats/attacks in the IoT environment has become an essential part of the operational ecosystem. The emerging technology is constantly evolving the IoT environment, which is leading to a change in security threats for automated networked systems.

AI methodologies are based on information-driven approaches wherein models are initially trained on various types of data to understand the attack’s behaviour^7^. There are different datasets for intrusion detection introduced in the literature, such as CI-CIDS 2017^8^, UNSWNB-15^9^ and ISCX-2012^10^ etc. However, none of these datasets is created under the consideration of the IoT environment. Recently, researchers started working on IoT datasets and introduced benchmark datasets for intrusion detection created under IoT environment, such as BoTIoT^11^ and DS20S^12^. However, features used in these datasets are insufficient and lack the newly proposed attack techniques. To overcome these problems, novel benchmark datasets such as LITNET-2020^13^ and IoTID20^14^ are presented recently. The LITNET-20 dataset is collected from Kaunas University of Technology (KTU) LITNET network and considers an intrusion into the academic network. In contrast, the IoTID20 dataset, sourced from home devices, is specifically curated for intrusion detection in smart devices, aligning closely with our research focus. It encompasses a diverse range of IoT attack types and their respective families. This study adopts the IoTID20 dataset for experimentation due to its strong relevance and comprehensive coverage within our problem domain. Key advantages of utilizing the IoTID20 dataset include its extensive collection of general attributes and features, which provide a rich dataset for training and evaluation. Additionally, the dataset offers a significant number of flow-based features, which are crucial for capturing dynamic network behaviors and patterns associated with IoT security threats. Moreover, each feature in the IoTID20 dataset is ranked, with a predominant emphasis on high-ranking attributes. This feature ranking enhances the dataset’s utility by prioritizing impactful variables that contribute significantly to the detection of IoT-related intrusions. By leveraging these attributes, the IoTID20 dataset not only facilitates robust experimentation but also ensures comprehensive coverage of IoT-specific security challenges. These factors collectively reinforce the dataset’s suitability and efficacy in validating our proposed intrusion detection methodology.

### Existing AI-based IDSs

The AI-based existing methodologies for network intrusion detection consider that data packet types and feature patterns in networks are the same as in an IoT environment. However, smart devices in an IoT environment are different in various aspects i.e. in terms of computational capability and functionality, characteristics of hardware, and ability to generate various data features. Therefore, IDS designed for network intrusion detection is not applicable to the IoT environment due to having distinct characteristics. Many researchers have proposed intrusion detection systems (IDSs) for IoT networks. The study in^15^ proposed an IDS using a support vector machine (SVM) for multiclass classification of attack types. In contrast, authors in^16^ claimed that the decision tree (DT) algorithm produced more powerful results as compared to SVM for IoT intrusion detection. Besides, ensemble approaches such as voting mechanism^17^, and random forest (RF)^18^ based solutions are proposed in the literature. Moreover, deep learning models such as long short-term memory (LSTM)^19^, convolutional neural network (CNN)^20^ and gated recurrent unit (GRU)^21^ are also introduced recently to improve the performance of IDS in IoT environment.

The existing ML and DL-based methodologies for IoT intrusion detection have several limitations. First, the performance of ML-based models relies heavily on the robustness of the employed handcrafted features, limiting their stability^5–7,22^. Second, their performance deteriorates when applied to big and high-dimensional traffic data containing different kinds of features coming through the IoT network^23,24^. Third, the learning capabilities of existing models for intrusion detection are limited to standard datasets without knowledge of the specific attacks^8,16,25^. Moreover, the existing systems cannot identify the attack, attack type and subtype of attack type simultaneously^26,27^. The IoTID20 dataset is highly imbalanced which tends the model to act aggressively towards the minority class^15^.

The objectives of this study are to improve IoT intrusion detection accuracy and identify its type and subtype by employing novel feature extraction techniques. This work proposes a hybrid CNN-GRU intrusion detection system for efficient feature extraction and classification of attack types and subtypes. Furthermore, training a less generalized model introduces model overfitting and produces biased results against minority classes. To overcome the problem of imbalanced data in the IoTID20 dataset, this work employs the latest feature-weighted synthetic minority oversampling technique (FW-SMOTE) to achieve class balance. The use of the IoTID20 dataset implies that the model is simulated on IoT network features in contrast to the literature work which majorly uses network domain datasets.

The hybrid approach of using CNNs and GRUs for detecting IoT attacks combines the strengths of CNNs in extracting spatial features from raw data with GRUs’ ability to capture temporal dependencies and patterns over time. This synergy allows the model to effectively analyze complex, high-dimensional IoT data streams, discerning both spatially distributed anomalies and temporal sequences indicative of attacks.

### Key contributions

The key contributions of the article are as follows.

- Innovative Hybrid Model: Introduction of a pioneering hybrid CNN-GRU model for IoT intrusion detection, demonstrating superior accuracy in predicting intrusion types and subtypes. This hybrid approach leverages the feature extraction capabilities of both CNN and GRU models, showcasing deep and intrinsic feature extraction from IoT intrusion samples.
- Comprehensive Evaluation: Rigorous experimental analysis conducted on two diverse datasets, IoTID20 and UNSW-NB15, representing IoT and network domains, respectively. The IoTID20 dataset, a recent addition in IoT datasets, includes labelled data for binary, type, and subtype intrusion attacks. UNSW-NB15, a balanced network domain dataset, encompasses labelled data for binary classification and intrusion attack types.
- Innovative Data Augmentation: Addressing the imbalanced nature of the IoTID20 dataset, the study employs Feature-Weighted SMOTE (FW-SMOTE), a cutting-edge variant of SMOTE. FW-SMOTE synthesizes data using Minoswaki distance with induced ordered weighted averaging (IMOWA), prioritizing features based on their relevance to the problem domain.
- Performance Benchmarking: The performance comparison of the proposed model is conducted with state-of-the-art DL architectures (CNN, RNN, LSTM, and GRU) and hybrid benchmark techniques (CNN-LSTM, TLBO-ELM, SS-DeepID and RNN-BPTT) from the literature. Our model outperforms in terms of accurately detecting the intrusion attacks, their types and subtypes with a trivial trade-off in detection time.

## Related work

As the field of IoT has emerged and deployed in various organizations, security analysts and researchers proposed numerous intrusion detection systems (IDSs) to identify threats in networks. These proposed methodologies often utilize various feature reduction and optimization techniques to enhance the efficiency and accuracy of IDS. The existing IDSs can be broadly categorised into ML-based and DL-based models.

### Machine learning-based intrusion detection systems

ML-based IDSs constitute a significant portion of the literature. For example, in^28^, the authors proposed three IDSs for IoT networks using DT, k-means clustering, and a hybrid approach combining DT and k-means clustering. The DT-based IDS achieved a 71–80% detection rate, k-means achieved a detection rate of 70–93% and the hybrid approach demonstrated a detection rate in the range of 71–75%. Apart from exhibiting an overall low detection rate, this work relies on randomly generated datasets for evaluation, and no publicly available IoT intrusion dataset was employed. In the study by^29^, an IDS was implemented using a three-layer approach based on supervised ML algorithms. The first layer identifies normal behaviour, the second layer detects anomalous behaviour, and the third layer identifies the type of attack. The model was validated using real-time network data from eight popular commercially available smart home devices, achieving F-measure values of 96.2%, 90.0%, and 98.0% for the three layers, respectively. However, the model was trained only for detecting a limited number of attacks.

Rashid et al.^30^, developed an ensemble approach using the DT, RF and extreme gradient boosting (XGBoost) classifiers for intrusion detection in IoT networks. They utilized the UNSW-NB15 and NSL-KDD intrusion datasets for performance comparison. The model achieved an accuracy of 89.90% on the NSL-KDD dataset and 93.7% on the UNSW-NB15 dataset. However, it only performs binary classification to identify intrusion samples from the IoT data stream, lacking the capability to detect attack types or subtypes. Saba et al.^26^ implemented a two-step hybrid approach for intrusion detection in an IoT environment. In the first step, a genetic algorithm-based approach is used for feature selection. In the second step, ML-based models such as DT, SVM and an ensemble classifier are implemented to identify attacks. The maximum accuracy of 99.8% was achieved with the ensemble approach, however, the proposed model was only evaluated for network intrusions. In contrast, Sarwar et al.^31^ used RF as a classifier in the proposed IDS. They employed particle swarm optimization (PSO) for feature reduction, and the XGBoost algorithm computed the value of the fitness function for PSO. The proposed model showed binary classification accuracy of 98% and 83% accuracy for multiclass classification. The model’s accuracy for multiclass classification is quite low which can be improved using advanced deep learning algorithms.

Similarly, the authors in^32^ proposed a two-phase approach for malicious traffic detection in computer networks. In the first phase, the proposed framework uses an MGA-SVM-based wrapper method. The wrapper method integrates the characteristics of two popular ML algorithms: multi-parent mutation and SVM. The second phase employs an ANN model for attack detection. The proposed IDS was evaluated on the publicly available NSL-KDD network dataset and it achieved an accuracy of 96.9%. The model shows a significant performance; however, intrusion attacks for IoT networks are not considered in this model. Indrasiri et al^33^ merged two different IoT datasets namely UNSW-NB15 and IoTID20 for intrusion detection from local network data streams and IoT-based traffic data. They employed an ensemble extra boosting forest (EBF) algorithm for the experimentation, yielding accuracies of 98.5% and 98% for binary and multiclass classification, respectively. However, the model is limited to detecting attacks, and the prediction of intrusion types is not implemented. The work by Maniriho et al.^34^ utilized the IoTID20 dataset to classify three IoT anomaly types: DoS attack, MITM, and Scan attack. The proposed model employs a hybrid technique for significant feature selection and an RF-based algorithm for the classification task. The IDS classifier exhibited improved performance with significant accuracy enhancements for identifying DoS (94.95%), MITM (94.97%), and Scanning (94.96%) anomalies. However, the classifier was not evaluated for the classification of subtypes within intrusion attacks.

Gandomi and Telikani in^35^ presented a cost-sensitive stacked auto-encoder (CSSAE) model for intrusion attack classification in IoT networks. In the first phase, CSSAE computes a cost matrix to determine the cost of each class based on its data distribution. The second step employs a two-layer AE model for feature learning to differentiate between majority and minority classes. The authors validated the proposed model using KDD CUP99 and NSL-KDD datasets for experimentation. The binary model detects the presence or absence of an anomaly while multiclass classification detects the exact attack type. The model performed better in detecting low-frequency attacks; however, the datasets used in the model are not purely IoT network datasets. Keserwani et al.^36^ introduced a random forest (RF)-based model to predict intrusions in IoT data streams. Feature reduction is performed using grey wolf optimization and PSO techniques. The three popular datasets known as NSL-KDD, CICIDS-2017 and KDDCup99 are used for performance validation. The average accuracy of the model was 99.66% for multiclass classification. However, the datasets used for performance evaluation are not exclusive representations of the IoT environment. In^37^, Hasan et al. proposed various ML-based IDSs for IoT networks using the DS2OS dataset. The ML techniques employed include logistic regression (LR), DT, RF, SVM, and ANN for prediction. The system achieved an accuracy of 99.4% for DT, RF and ANN while it was 98.2% and 98.3% for SVM and LR respectively. However, the provided results are demonstrated on a small dataset and similar performance is not guaranteed for bigger datasets.

### Deep learning-based intrusion detection systems

Recent studies showed that deep learning-based IDSs tend to exhibit superior performance in detecting attacks in IoT networks. For example, researchers in^38^ introduced an intrusion detection framework for IoT networks employing three deep learning approaches: CNN, LSTM, and a hybrid model combining CNN and LSTM named CNN-LSTM. The framework was tested on the IoTID20 dataset and achieved the best accuracy with LSTM (99.20%), followed by the hybrid CNN-LSTM model (98.0%) and CNN (96.60%). However, the study uses an imbalanced dataset, and balancing it could yield even better results. Another study by Alqahtani in^39^ proposed a novel hybrid optimized LSTM approach where CNN extracts the spatial and temporal correlated features from the IoT dataset, and LSTM is used for predicting intrusion attacks. The model incorporates the firefly swarm optimization technique for feature selection, reducing the computational overhead. Evaluation on UNSW-NB15 and NSL-KDD, two popular network intrusion datasets, demonstrated the deep learning model’s superior performance with a prediction accuracy of 98.89%. However, it struggled to identify new types of anomalies in high network traffic streams. Moreover, when assessed on real-time IoT datasets, the model experienced longer training times, diminishing its overall capability.

Similarly, Abdel-Basset et al.^40^ presented a multiscale residual temporal convolution module in their semi-supervised DL approach for intrusion detection (SS-Deep-ID), This module is designed for learning spatiotemporal features and incorporates an attention mechanism to extract localized features. The proposed SS-DeepID model is evaluated on two network intrusion datasets, CI-CID2017 and CI-CID2018, demonstrating improvements in efficiency and accuracy. However, the model takes more time to identify intrusion attacks in real-time data traffic datasets. Xiao et al.^41^ employed two dimensionality reduction techniques, namely PCA and autoencoder (AE) to assess the impact of a reduced feature set on the classification of intrusion attacks. The resulting feature set is then fed to a CNN model for attack classification. The model is tested on the standard KDD-CUP99 dataset, revealing that AE (0.940) exhibited better accuracy than PCA (0.930). However, the detection accuracy of the model is relatively low compared to other existing models. Conversely, Safiullah et al.^42^ proposed an intrusion detection framework using a deep CNN in the binary and multiclass categories. The framework is tested on the IoTID20 dataset with varying batch sizes, and performance evaluation is conducted using standard metrics. The model achieves high accuracy, F1-score, precision and recall of 99% for binary classification but lower values ranging from 70 to 97% for multiclass classification. However, the proposed model’s performance comparison is not carried out with the related studies in the provided context.

Furthermore, Diro et al.^43^ work presents a DL model for intrusion detection and type prediction in IoT networks. The model was tested using the NSL-KDD network intrusion dataset and its performance analysis was conducted with traditional ML-based IDSs. The model implements two approaches: centralized detection and distributed detection. Results indicate that the accuracy of the distributed attack detection approach outperforms the centralized detection approach. The authors provide empirical evidence supporting the assertion that the DL model is better suited for intrusion detection than shallow networks. However, the proposed DL-based IDS was not tested on real IoT datasets. Moreover, it only considered a limited number of attack types for model validation. Meanwhile, Qaddoura et al.^44^ presented a hybrid solution for IoT network intrusion detection using a simple feedforward neural network and LSTM models. The model employed the SMOTE oversampling technique to resolve the class imbalance issue in the training dataset. The performance analysis involved comparing results with other ML classifiers such as KNN, Naive Bayes etc. The G-mean metric of the proposed system was 78% for SLFN while it was 75% for KNN and less than 50% for the rest of the models.

### Hybrid deep learning-based intrusion detection systems

The study by Ullah et al.^45^ proposed a set of hybrid deep learning models designed for intrusion detection in IoT networks. The proposed hybrid models include CNNLSTM, CNNBiLSTM, and CNNGRU. The authors performed a feature selection procedure using the PSO algorithm. The system was tested on seven datasets, i.e., NSLKDD, MQTT, IoT-NI, BoT-IoT, MQTTset, IoT-23 and IoT-DS2. The performance analysis was conducted for both binary and multiclass scenarios and proposed hybrid models outperformed existing models across all evaluation metrics. Dushimimana et al.^46^ introduced a bidirectional recurrent neural network (BRNN)-based IDS for IoT networks. The model performance analysis was conducted with RNN and gated recurrent neural network (GRNN). The study utilized 10% of the original KDD dataset for model validation, and the proposed BRNN demonstrated the highest accuracy of 99.04% compared to other existing models. However, the BRNN model is evaluated on a small dataset and performance on real-time high-traffic data still needs to be evaluated. Similarly, the work by Latif et al.^47^ proposed an IDS based on a random neural network for industrial IoT. The model was evaluated using the DS2OS dataset. The model was evaluated using the DS2OS dataset and achieved an accuracy of 99.20%, surpassing existing ML and DL models such as ANN, SVM, and DT. However, the selected dataset is severely imbalanced with the DOS attack having 5780 samples, while the rest of the attacks have fewer than 1600 samples.

A study in^48^ implements a transformer-based intrusion detection approach, named IDS-INT, using hybrid deep learning model CNN-LSTM. The model is applied to three network domain datasets: UNSW-NB15, CIC-IDS2017, and NSL-KDD. First, a BERT transformer-based transfer learning approach is used to learn feature representation from the network traffic data. Then, CNN-LSTM is used to extract deep features from the learned feature representation. The model achieved average accuracies of 98.81% on UNSW-NB15, 99.15% on CIC-IDS2017 and 98.17% on the NSL-KDD dataset. However, this study focuses on network domain datasets for intrusion detection and exclusively performs multiclass classification providing no specific details about attack types and subtypes. Another hybrid model proposed by Alsudani at al.^49^ uses multiple ML algorithms, including extreme learning machine (ELM) and teaching learning-based optimization (TLBO) for intrusion detection in computer networks. The model achieves an accuracy of 98.21% accuracy on the NSL-KDD dataset. However, the model only detects intrusion in the network domain datasets without providing predictions for attack types or subtypes. Furthermore, the model accuracy is quite low and could potentially be enhanced by employing hybrid deep learning models.

In response to the increasing network security vulnerabilities in the cyber environment, a hybrid deep learning-based network intrusion detection system (NIDS), named DCNNBiLSTM is proposed^50^. The NIDS achieves high accuracy rates of 100% and 99.64% on CICIDS2018 and Edge IIoT, real-time traffic datasets. By combining the strengths of different deep learning models, the system accurately detects irregular attack patterns, outperforming single models in most scenarios. However, the proposed model is simulated on datasets with limited attack types and predicting subtypes of attacks is not taken into consideration. Finally, achieving 100% accuracy may indicate the potential case of overfitting. More recently, Saba et al.^51^ proposed a CNN-based approach for binary classification of intrusion attacks. The model was tested using NID and BoTIoT datasets and achieved accuracies of 99.51% and 92.85%, respectively. However, this study is only focused on binary classification and achieves low accuracy in comparison with other studies.

A study by Hussain and Hnamte^52^ explores the implementation of a DL approach on SDN using a DNN model to monitor network activity and detect malicious traffic. The study successfully addresses security concerns, achieving a high accuracy rate. However, further work is needed to enhance the robustness of the model and evaluate the feasibility of using deep learning in production environments. Moreover, the proposed model does not address the assessment of its impact on network latency and performance in practical SDN setups. Haideri and Jamali^53^ and Alsoufi et al.^54^ present systematic literature reviews (SLRs) of the intrusion detection models in IoT networks. These studies provide an in-depth and systematic analysis of existing models offering taxonomies, reviews, and trends related to IoT intrusion detection. Furthermore, they highlight the advantages and drawbacks of the published techniques and propose future research directions.

Research in IoT intrusion detection systems (IDSs) has demonstrated that both machine learning (ML) and deep learning (DL) approaches offer promising solutions, each with unique strengths and challenges. ML-based IDSs, employing algorithms like Random Forest (RF) and Support Vector Machines (SVM), achieve high accuracy but face limitations in real-world IoT deployments. These limitations arise from their reliance on datasets not specifically designed for IoT contexts and the burden of manual feature extraction. Additionally, the complexity of IoT network behaviors and dataset restrictions hinder these models from detecting specific IoT-related attack types, often restricting them to binary or multiclass classification of attacks. Conversely, DL-based IDSs, utilizing models such as Convolutional Neural Networks (CNN) and Long Short-Term Memory (LSTM) networks, excel at identifying complex patterns in IoT traffic. DL models adapt better to the diverse and dynamic nature of IoT environments, automatically learn relevant features from raw data, and handle large-scale data more efficiently, which is crucial given the vast amounts of data generated by IoT devices. However, DL approaches also come with drawbacks, such as higher training times and increased data volume requirements. This research aims to address these gaps by developing more robust DL models tailored specifically for IoT environments. A summary of key findings reveals that while DL models offer significant advantages in adaptability, feature learning, and scalability, there remains a need for further refinement to overcome their inherent challenges and enhance their applicability in real-world IoT scenarios.

## Proposed methodology

**Fig. 1:**
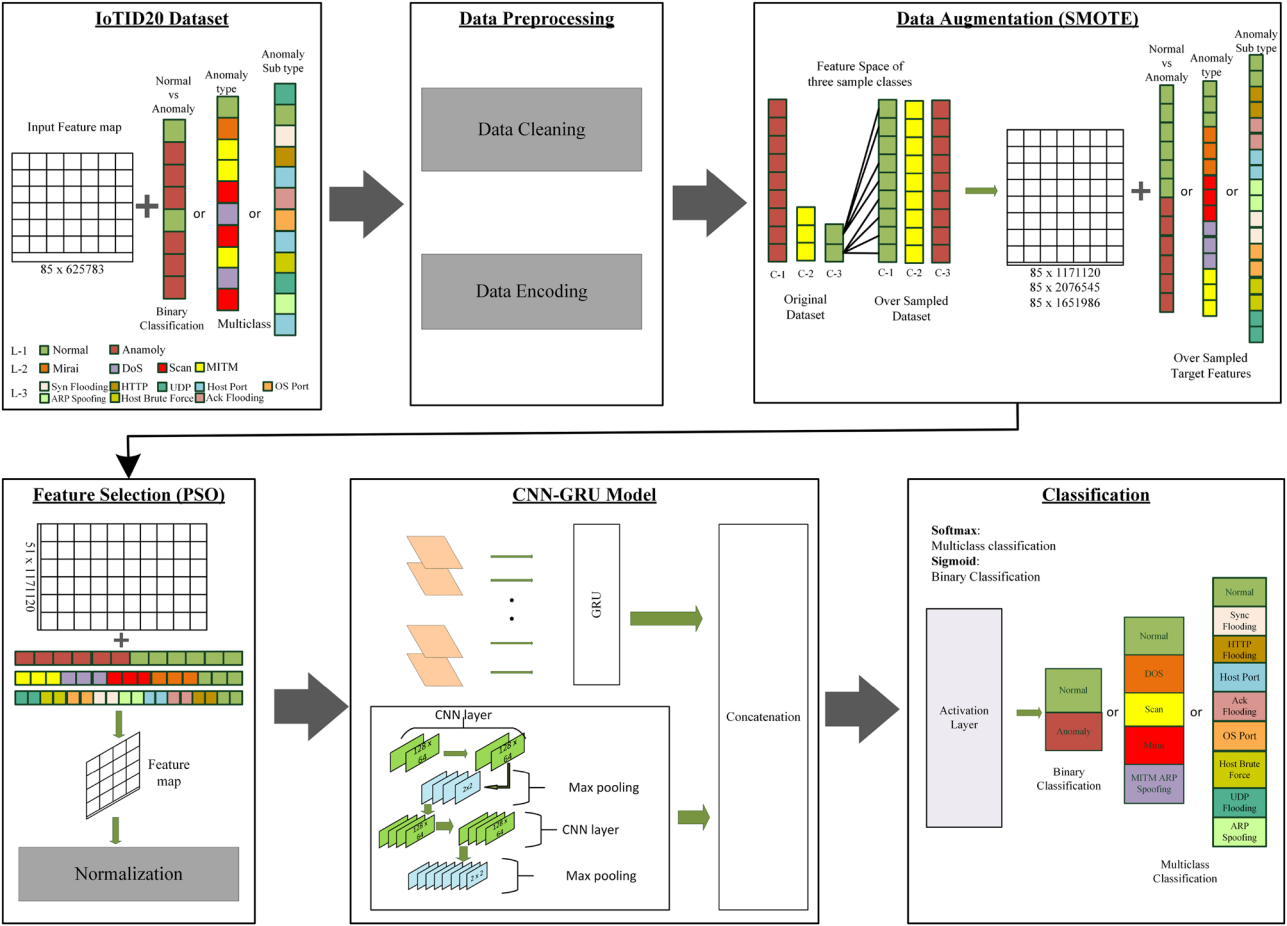
Block diagram of the proposed model for intrusion detection in IoT networks.

An illustration of the overall model working is presented using a block diagram in Fig. 1. The first step involves acquiring the IoTID20 dataset and applying exploratory data analysis (EDA) techniques. The model then applies preprocessing operations and summarises the main characteristics of the dataset using data visualisation methods. Further, it implements data oversampling to overcome the problem of an imbalanced dataset. Subsequently, the feature selection is applied which removes unnecessary features from the dataset. The complex and hidden features are extracted using the proposed hybrid CNN-GRU model. The resultant feature vector is fed to the fully connected layer for prediction of the intrusion attack, its type or subtype. The subsequent sections elaborate on individual components of the proposed model.

### Exploratory data analysis

#### Data preprocessing

**Fig. 2:**
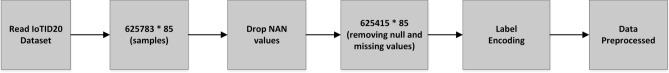
Block diagram of the data preprocessing steps used in the model.

The data preprocessing steps are highlighted in Fig. 2. These operations are required to make the input dataset compatible with ML and DL models. Thus, we followed the process of data cleaning and encoding which are two major steps of preprocessing.

In our initial step, we performed data cleaning, discarded the useless indices, and removed the missing values and NaN values. The features of the data type “object” are converted into a float data type to avoid errors. After data cleaning, target variables are encoded according to their class types. The encoded values of 1 and 0 are used for attack and normal class samples respectively. For multiclass classification of the attack type, Mirai, DoS, Scan, Normal and MITM are encoded as 1, 2, 3, 4, and 5. For the multiclass classification of attack subtype, Mirai-Ackflooding, DoS-Syncflooding, Scan-PortOS, Mirai-Hostbruteforce, Mirai-UDPFlooding, Mirai-HTTPFlooding, Normal, Scan-Hostport, MITM-ARPSpoofing are encoded as 1, 2, 3, 4, 5, 6, 7, 8, and 9 respectively. It has been observed during preprocessing that the IoTID20 dataset is highly imbalanced. To overcome this issue, FW-SMOTE is used to perform data augmentation for the minority classes.

#### Data augmentation

Imbalance classes of the IoTID20 dataset tend the model to aggressively classify the minority classes/labels as majority class. Consequently, it degrades the performance of the classification model and produces more false positives. It is challenging for practitioners and researchers to avoid biased predictions in favour of the majority class due to a low number of minority class samples.

There are many techniques proposed to overcome the imbalanced dataset problem and oversample the minority class instances. The SMOTE is a well-known and one of the most commonly used oversampling techniques in the past few years^55^. It considers each of the minority class samples from the feature space, finds one of their k-nearest neighbours and generates synthetic samples by linearly interpolating between samples and their selected neighbours. This work employs a recent variant of SMOTE, known as feature-weighted SMOTE (FW-SMOTE), which introduces weights with the features using the induced ordered weighted averaging (IOWA) operator while calculating distance with the neighbours. Unlike SMOTE which uses Euclidean distance, FW-SMOTE employs Minkowski distance for finding neighbour samples^56^. Thus, the FW-SMOTE oversampling method prioritizes features from the feature space in the neighbour selection process and improves the quality of synthetically generated minority class samples.

Algorithm 1 describes the working of the FW-SMOTE algorithm for IoTID20 data augmentation. Steps 1 and 2 explain the input and output parameters of the algorithm respectively. In step 3, the original set of minority samples in *T* is used to initialise the oversampled minority class set T*. The order-inducing variable *u* needed by induced Minkowski ordered weighted averaging (OWA) distance (IMOWAD) metric is then calculated using the feature ranking approach in step 4. In step 5, the order-inducing variable *u* is used to derive the weights for the IMOWAD distance. A regular increasing monotone (RIM) quantifier with input parameter $\alpha$ is used for this phase. Step 6 implements the SMOTE’s feature selection strategy, by defining a subset *S*. Step 7 traverses over all the minority samples in T, and uses the IMOWAD function to calculate the neighbourhood of size *k* (see steps 8 to 10). From subset *S*, ${x}_{k}$ random samples are selected in step 12 and step 13 interpolates the target sample to produce synthetic samples ${x'}_{k}$. At step 14, the synthetic samples ${x'}_{k}$ is added to T*, and then in step 15, a randomly chosen sample ${x}_{k}$ is omitted from *S*. Every minority class goes through this method *N* times until balanced samples are generated.

**Algorithm 1:**
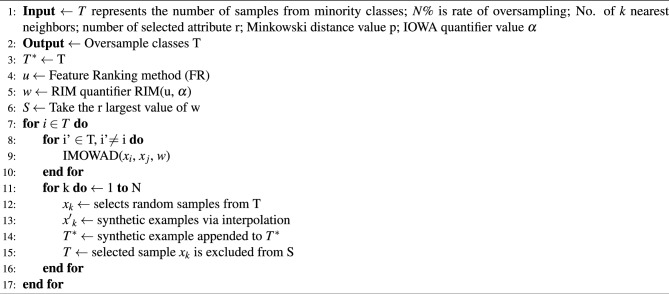
FW-SMOTE for oversampling of IoTID20 dataset^56^

**Fig. 3:**
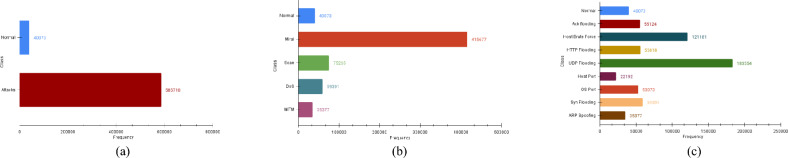
IoTID20 dataset histogram analysis for binary, attack types and subtypes. The class imbalance problem due to the highly varied number of samples for different attack types is visible from the description.

In this study, three different experiments are performed, and in each of the three cases, a dataset imbalance issue is encountered. Fig. 3 highlights the data imbalance issue using histogram analysis and presents the count of samples for various attack types and their subtypes. It can be seen that the total number of attack samples in the IoTID20 dataset is 5,85,710 and normal traffic data samples are 40,073. Similarly, for attack types, such as the Mirai attack has 4,15,677 samples, Scan attack samples are 75,265, DoS attack has 59,391 samples and MITM sample count is 35,377. For the attack subtypes, the sample count is AckFlooding (55,124), HostBruteForce (121,181), HTTPFlooding (55,818) and UDPFlooding (183,554). Similarly, HostPort subtype has 22,192 samples, OSPort (53,073), SynFlooding (59,391) and ARP Spoofing (35,377).

**Fig. 4:**
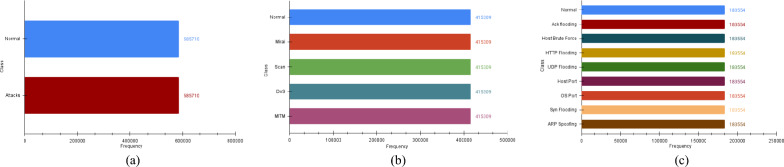
IoTID20 dataset histogram analysis for normal, attack types and subtypes after applying data preprocessing and data augmentation operations.

The first case for data balancing is the binary classification between normal data samples and intrusion attacks. The sample breakdown demonstrates that the IoTID20 dataset attacks samples represent 94% population of the dataset. On the other hand, normal samples cover only 6% part of the dataset. Thus, it is an imbalanced dataset for binary classification of attacks. After applying the FW-SMOTE technique, binary samples for normal versus attack are balanced as 50% samples representing each activity. Consequently, the number of samples for both attacks and normal classes becomes 585,710 and Fig. 4a displays the histogram of the resultant distribution.

The second data imbalance scenario arises in identifying intrusion type. Figure 3 shows that the IoTID20 dataset contains only 5.7% of the MITM intrusion samples representing a minority class of the dataset. In comparison, Mirai intrusion comprises 66.47% of the dataset and represents the majority class of the dataset. Thus, the dataset is imbalanced for the multiclass classification. So, to overcome this issue, the FW-SMOTE oversampling technique is applied and minority classes are oversampled and balanced with the majority class. After over-sampling, each type class has 415,309 samples. The balanced data distribution is given in Fig. 4b.

In the third case of our study, we faced class imbalance issues in the multiclass classification task of the intrusion subtype. As shown in Fig. 3, Host Port subtype has only 22,192 samples which constitute only 3.5% of the total dataset. On the other hand, UDP Flooding consists of 183,554 data samples representing 29.3% of the total dataset. The same goes with other subtypes where the sample count is far lower than the UDP Flooding. It highlights an imbalanced dataset scenario which will negatively impact the performance of IDS. Hence, the dataset needs to be balanced for attack subtype samples. After applying the FW-SMOTE technique, minority classes are oversampled to create a balanced dataset and the percentage ratio is 11.1% for all subtypes as shown in Fig. 4c.

### Feature selection

The IoTID20 dataset contains features that hinder model generalisation and exhibit no discernible impact on accurately classifying the target class. Particle swarm optimization (PSO) is a widely used feature selection technique to address model overfitting and computational complexity problems^57^. It takes an initial population of particles each representing a subset of features, and iteratively refines these subsets to identify optimized feature combinations. The optimisation is achieved using an objective function which measures solution fitness with the target solution.

Algorithm 2 describes the working of the PSO algorithm in the context of feature selection with the IoTID20 dataset. Initially, it chooses *N* as the particle population size and randomly assigns both the position and velocity for each particle. The position and velocity determine the state of the particle within the solution space.

1 $$\begin{aligned}&p_i = \{p_{i1},p_{i2}, p_{i3}, . . . p_{iS} \} \end{aligned}$$

2 $$\begin{aligned}&vp_i = \{v_{i1},v_{i2}, v_{i3} . . . v_{iS} \} \end{aligned}$$

where *S* represents the overall search space of the particle. The swarm optimizer evaluates the fitness value of particles and keeps a record of the two best values of particles in each iteration. The first value, ${p}_{best}$ represents the local best fitness value achieved by each particle and the second value ${g}_{best}$, represents the best fitness value achieved by any particle globally. The updated value of velocity and position is computed using Eqs. (3) and (4), respectively.

3 $$\begin{aligned}&v^{j+1}_{id} = w * v^{j}_{id} + c_1 * rand_{1i} ( P_{id} - p^{j}_{id} ) + c_2 * rand_{2i} * ( P_{gd} - p^{j}_{id} ) \end{aligned}$$

4 $$\begin{aligned}&p^{j+1}_{id} = p^{j}_{id} + v^{j+1}_{id} \end{aligned}$$

where *d* denotes the dimension and *j* represents the iteration variable over the search space. The *w* denotes the inertia weight while the acceleration is represented using constants $c_1$ and $c_2$. The $P_{id}$ and $P_{gd}$ are the ${p}_{best}$ and ${g}_{best}$ values in the search space respectively and ${rand}_{1i}$ and ${rand}_{2i}$ are the random values distributed between 0 and 1^38^. The iterative process continues until best-defined criteria or targeted values are attained.

**Algorithm 2:**
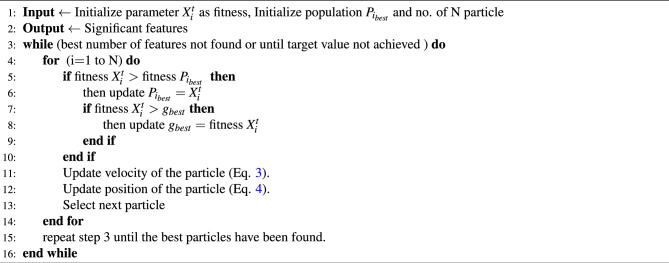
Principal swarm optimization (PSO) technique for feature selection in IoTID20 dataset^38^

**Fig. 5:**
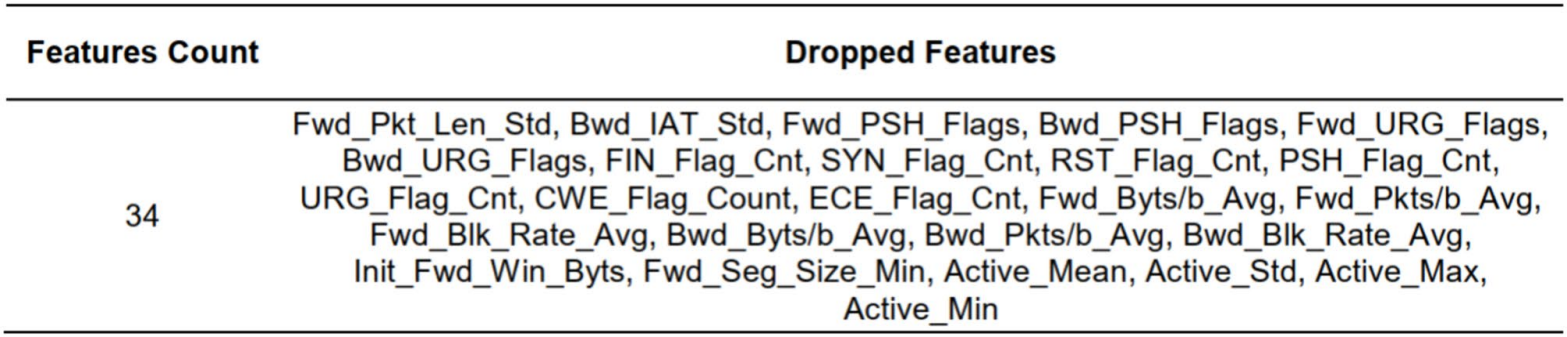
Dropped features from IoTID20 dataset after applying particle swarm optimization (PSO) technique.

**Fig. 6:**
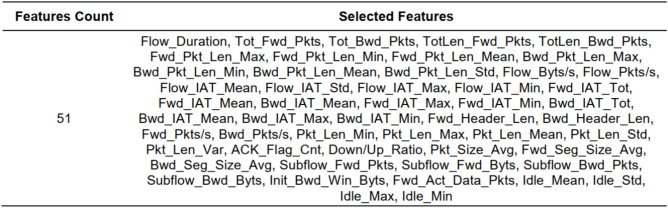
Selected features from IoTID20 dataset after applying particle swarm optimization (PSO) technique.

Figure 5 provides the list of dropped features from the dataset. A total of 34 features are found to be less significant with a low correlation score observed between these features and both normal and attack features. Hence they are not considered for the training dataset and are excluded from the feature set employed for model training. Only 51 significant features are selected for the training and testing phases of the proposed method. The list of selected features is provided in Fig. 6

### Feature extraction

**Fig. 7:**
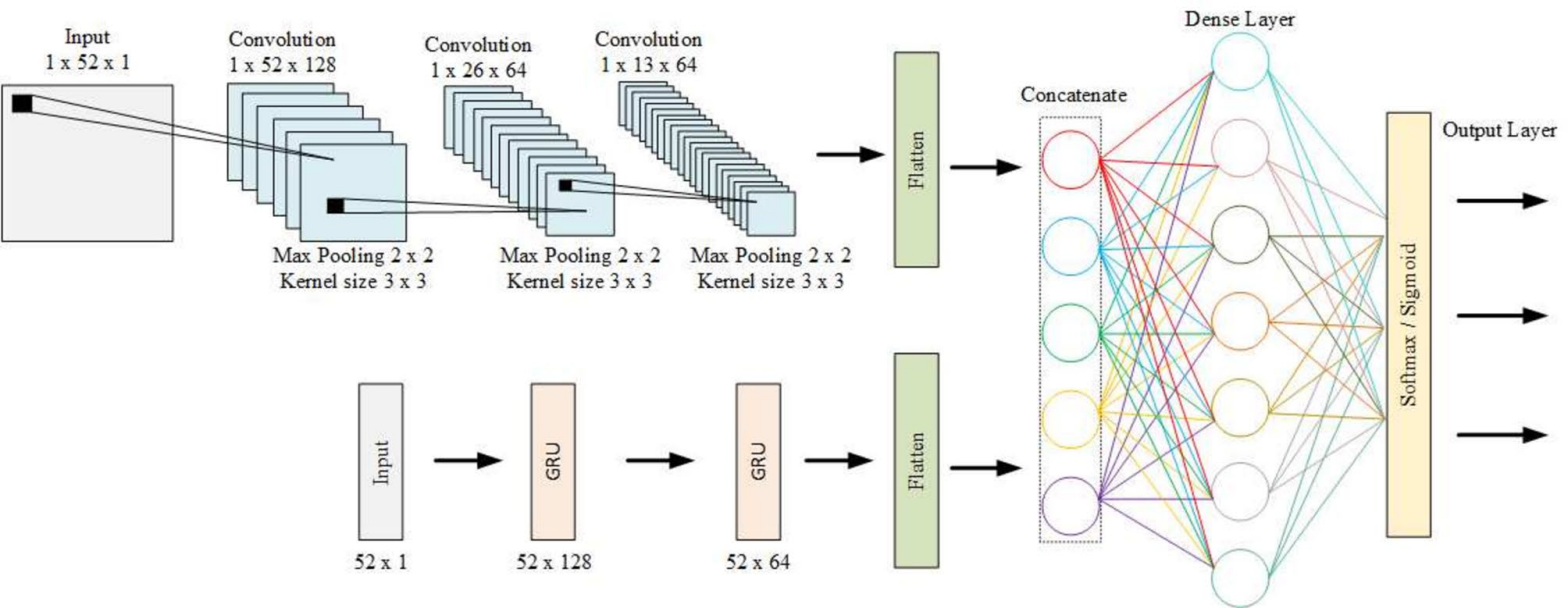
Layered diagram of the proposed hybrid CNN-GRU model.

The proposed model uses a double-branch hybrid CNN-GRU model for feature extraction. CNNs^58^ are widely used for extracting dominant and latent abstract features from the original input dataset. It consists of a sequence of convolutional and pooling layers to transform the input data into the target output data. The architecture of the proposed hybrid CNN-GRU model is given in Fig. 7.

In this study, we used CNN to extract higher-order features and local features of IoT activities. The designed network comprises two convolutional layers and two max-pooling layers. The convolution operation is performed using Eq. (5).

5 $$\begin{aligned} Z = X \otimes F \end{aligned}$$

where *X* denotes the input feature vector and *F* represents the number of convolution layers. The convolution layer utilizes three kernels and a set of 100 convolution filters. Subsequent to each convolution operation, a batch normalization operation is performed, which is defined using Eqs. (6), (7) and (8):

6 $$\begin{aligned}&\mu = \frac{1}{m} \sum _{i=1}^{m} Zi \end{aligned}$$

7 $$\begin{aligned}&\sigma = \frac{1}{m} \sum _{i=1}^{m} Zi - \mu \end{aligned}$$

8 $$\begin{aligned}&BN(\hat{Zi}) = \frac{Zi-\mu }{\sqrt{\sigma ^2+\varepsilon }} \end{aligned}$$

where $\mu$ denotes mini-batch mean operation, $\sigma$ computes variance and $BN(\hat{Zi})$ signifies the final batch normalization outcome applied on the convolved feature vector *Z*. To reduce the dimension of newly formed complex features, a max-pooling layer with two kernels is introduced. An activation function ReLU is employed to transmit the data, choosing the maximum value from the pooled feature vector.

9 $$\begin{aligned} ReLU = max(\hat{Zi}) \end{aligned}$$

We also fed the input feature vector from the input layer to the GRU model. GRU, an extension of standard recurrent neural networks^59^. represents a simplified version of LSTM^60^ featuring two gates - reset and update. The model retains information if it is considered useful; otherwise, it is discarded. The reset and update gates are defined in Eqs. (10) and (11).

10 $$\begin{aligned}&r_t = \sigma (W_r \cdot [h_{t-1}, X_t]) \end{aligned}$$

11 $$\begin{aligned}&z_t = \sigma (W_z \cdot [h_{t-1}, X_t]) \end{aligned}$$

where $W_r$ and $W_z$ denote the weights of reset and update gates respectively. $X_t$ represents the input vector and $h_{t-1}$ signifies the previous hidden state. The current state of the updated memory is defined using Eqs. (12) and (13):

12 $$\begin{aligned} \tilde{h}_t = \tanh (W_h \cdot [r_t \odot h_{t-1}, X_t]) \end{aligned}$$

13 $$\begin{aligned} h_t = (1 - z_t) \odot h_{t-1} + z_t \odot \tilde{h}_t \end{aligned}$$

The primary advantage of using the GRU layer lies in its utilization of fewer parameters during training, leading to faster execution, efficient learning and reduced memory consumption. Moreover, GRU deeply examines the contextual information and relational aspects of feature patterns. The proposed model uses two GRU layers each consisting of 128 memory units. The outputs from CNN and GRU models are flattened and concatenated to create a hybrid model.

Algorithm 3 outlines the operation of the proposed hybrid CNN-GRU model. It takes a dataset with features *X* and labels *Y* as input. The number of CNN layers, *F*, and GRU units, *U*, are also specified as inputs. The algorithm produces standard ML metrics accuracy, precision, recall and F1-score as output. The CNN and GRU parameters are initialised, and the dataset is split into training and testing sets. The two functions, CNN and GRU, define the operations of the CNN and GRU branches of the model, respectively. The CNN function, defined in steps 6 to 15, takes an input tensor *‘X‘* and applies convolution, batch normalization, activation, and pooling operations to extract the feature vector. The GRU function, defined in steps 16 to 25, takes an input tensor *‘X‘* and iteratively applies the GRU operations to compute the final hidden state $h_t$. The GRU equations from steps 19 to 22 depict the updating process of the hidden state and memory cell in each iteration *t*.

The number of CNN layers determines the depth of feature extraction from the input data. Deeper networks can capture more complex patterns and hierarchical representations. Choosing an appropriate depth depends on the complexity of the dataset and the desired level of abstraction. We used empirical studies and prior research to guide us about the choice of hyperparameters which are mentioned in Table 2. The benchmarks on similar tasks suggested a typical range of layers that achieve good results. GRUs are chosen here likely due to their effectiveness in modeling sequential data and capturing long-range dependencies. These units affects the model’s capacity to remember and utilize sequential information effectively.

**Algorithm 3:**
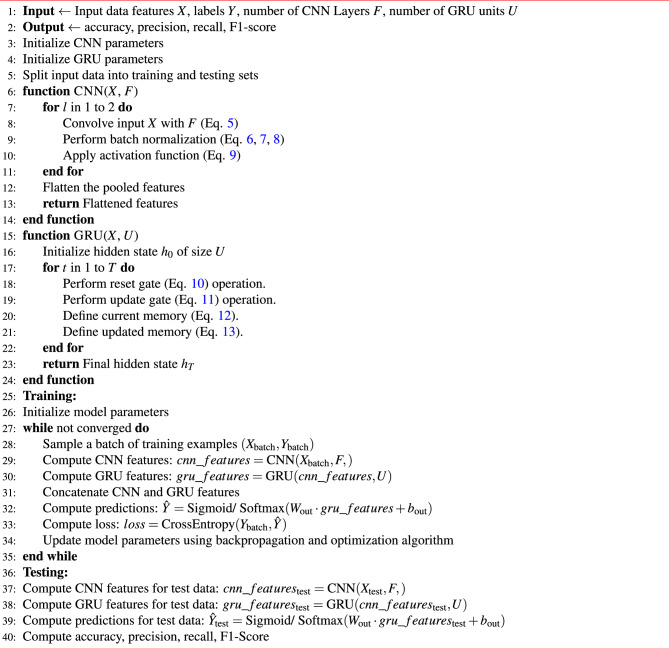
Proposed CNN-GRU hybrid model.

The main algorithm comprises a training loop that samples batches of training examples and computes CNN and GRU feature vectors (steps 29 to 31). Subsequently, it concatenates these vectors (step 32), performs predictions (step 33), calculates the loss using cross-entropy (step 34) and updates the model parameters through backpropagation and optimization (step 35). The sigmoid and softMax activation functions are employed for binary classification and multiclass classification tasks, respectively. Sigmoid determines whether it is an intrusion attack or a normal sample while attack type and subtype are identified using the SoftMax activation function. The algorithm continues to iterate until the convergence criteria are met.

We split the data into training and validation sets and used the validation set to monitor the model’s performance during training. This helps detect overfitting and ensures that the model generalizes well to unseen data. We monitored the decrease in the loss function and tracked metrics like accuracy, precision, recall, and F1-score on the validation set to gain insights into how well the model generalizes to new data and whether it is meeting performance goals. The stability of the metrics indicated that the model has converged, and further training may not yield significant improvements.

After training, the algorithm proceeds to the testing phase, where it computes CNN and GRU feature vectors for the test data (steps 38 and 39), makes predictions using the learned parameters (step 40), and evaluates the accuracy, precision, recall, and F1-score metrics. The proposed model is evaluated for three cases: (1) binary classification for normal data versus intrusion detection, (2) multiclass classification for the type of intrusion attack and (3) multiclass classification for a subtype of intrusion attack.

## Experimental settings

The implementation and training of the deep learning models are conducted using Kaggle GPUs and Python 3.8. TensorFlow and Keras, both open-source libraries, are used to build deep learning models. The scikit-learn library is used to generate the confusion matrices and performance evaluation metrics.

### Datasets

The experiments are performed on the IoTID20 dataset, specifically designed for IoT intrusion detection considering the IoT environment. This dataset is a balanced version derived from the original IoTID20 dataset, created through the FW-SMOTE-based data augmentation approach. The target dataset encompasses 51 IoT network features and three target label features. The feature selection method outlined in “Feature selection” section selects the 51 relevant features while discarding the irrelevant ones. The three label features each describe the binary, attack types and subtypes scenarios. The dataset is partitioned into 80% for training purposes and 20% for testing the model.

UNSW-NB15 dataset from the network intrusion detection domain is used to validate the proposed model. UNSW-NB15 dataset consists of 10 different types of network attacks. It is a balanced dataset and only contains features and labels that are suitable for detecting an attack and its type. Therefore, we compare the results for these two case scenarios only. UNSW-NB15 dataset contains 37 features after prepossessing and two target features; attack and attack type. The dataset is divided into 80% for training and 20% for testing to evaluate the performance. The specifics of the dataset distribution into training, validation and testing sets are provided in Table 1

**Table 1:** Dataset description of IoTID20 and UNSW-NB15 datasets used in this study. Data distribution details are also provided for training, validation and testing sets.

Dataset	Feature count	Dataset domain	Publicly available	Label type	Class count	Training	Validation	Testing
IoTID20	51	IoT	Yes	Binary attack	2	937,136	117,142	117,142
				Attack type	5	1,661,236	207,655	207,655
				Attack subtype	9	1,321,583	165,199	165,199
UNSW-NB15	37	Network	Yes	Binary attack	2	2,032,037	254,004	254,004
				Attack type	10	2,032,037	254,004	254,004

### Hyperparameters

The hyperparameter tuning of the models is performed based on insights from the literature review and through a grid search mechanism following multiple trial experiments. Table 2 specifies the selected values of different hyperparameters for this study. The training is executed for 10 epochs for the binary classification case and 30 epochs for the multiclass classification. A batch size of 64 is defined for each case, namely, binary and multiclass classification. The learning rate is set as 0.001 for both binary and multiclass scenarios. Dropout rates ranging from 0.2 to 0.4 are defined to mitigate overfitting during model training. The Adam optimizer is employed for individual deep learning models, while Nadam is applied for hybrid models, including the proposed model. The sigmoid and softmax are activation functions for binary and multiclass classification scenarios respectively. Similarly, binary_crossentropy and categorical_crossentropy serve as loss functions for binary and multiclass classifications, respectively.

Ten epochs were selected because binary classification tasks require fewer epochs compared to multiclass classification due to the simpler decision boundary. Ten epochs provide sufficient iterations for the model to learn patterns in the data without risking overfitting. Multiclass problems often involve more complex decision boundaries and require more epochs for the model to learn to distinguish between multiple classes accurately. Thirty epochs allow the model to converge more thoroughly on the training data. A batch size of 64 strikes a balance between computational efficiency and gradient accuracy. A value of 0.001 for learning rate is commonly chosen as a starting point for many deep learning models. It is small enough to ensure stable convergence and prevent overshooting the optimal solution. The range of dropout rates from 0.2 to 0.4 suggests a moderate to aggressive regularization approach, balancing between model complexity and overfitting mitigation based on the complexity and size of the dataset. Nadam incorporates the Nesterov momentum, enhancing gradient descent with faster convergence and potentially better generalization performance in hybrid models like CNN-GRU, where both convolutional and recurrent layers are involved.

**Table 2:** Hyperparameter settings of implemented deep learning models.

Hyperparameters	CNN	RNN	LSTM	GRU	CNN-LSTM	Proposed (CNN-GRU) model
Input neuron	256	128	128	128	128	128
Hidden neuron	128	–	–	–	64	64
Epochs	10–30	10	10-30	10-30	10-30	10-30
Optimizer	Adam	Adam	Adam	Adam	Nadam	Nadam
Dropout rate	0.3	0.4	0.4	0.2	0.2	0.2
Batch size	64					
Learning rate	0.001					
Loss	Binary_Crossentropy, Categorical_Crossentropy					

### Evaluation metrics

The assessment of the proposed IDS involves the use of standard evaluation metrics as specified in Eqs. (14), (15), (16), and (17). The accurate prediction of attack and normal samples is represented by true positive (TP) and true negative (TN) parameters, respectively. Whereas inaccurate classification of normal and attack instances are indicated by the false positive (FP) and false negative (FN) values, respectively^61^.

The percentage of correctly identified occurrences, both true positives and true negatives, out of all the examples analysed is known as accuracy. Out of all instances predicted as positive (true positives and false positives combined), precision is the percentage of true positive predictions (incorrectly identified intrusions). The recall metric quantifies the percentage of accurately detected intrusions, or true positive predictions, across all positive cases, including false negatives and true positives. The harmonic mean of recall and precision is the F1 score. These metrics are given by;

14 $$\begin{aligned}&Accuracy = \frac{(TP+TN)}{(TP+TN+FP+FN)} \end{aligned}$$

15 $$\begin{aligned}&Precision = \frac{TP}{(TP+FP)} \end{aligned}$$

16 $$\begin{aligned}&Recall = \frac{TP}{(TP+FN)} \end{aligned}$$

17 $$\begin{aligned}F1{\text{-}}score = \frac{2*Precision*Recall}{Precision+Recall} \end{aligned}$$

False positives in intrusion detection, which mistakenly label routine activities as intrusions, as well as false negatives, which miss real incursions, can have serious repercussions. Metrics like precision, recall, and F1 score are therefore critical since they evaluate the system’s capacity to effectively identify and categorise network intrusions while reducing errors. By assisting security analysts and system administrators in evaluating the IDS’s dependability and efficacy in practical situations, these metrics enable them to make decisions about optimising the model and enhancing network security posture.

The accuracy, precision, recall, and F1 score are essential for assessing how well intrusions are detected. The IDS’s total accuracy in categorising events as either normal or intrusive is represented by its accuracy. Precision quantifies the percentage of intrusive instances that the intrusion detection system (IDS) actually flags. This indicates the IDS’s capacity to minimise false positives, which is important for lowering the number of needless alarms and alert fatigue. Contrarily, recall quantifies the percentage of real intrusive incidents that the IDS successfully detects, demonstrating its capacity to identify every incursion and prevent false negatives–two essential components of guaranteeing thorough security coverage.

## Results evaluation and performance analysis

### Binary attack classification

**Fig. 8:**
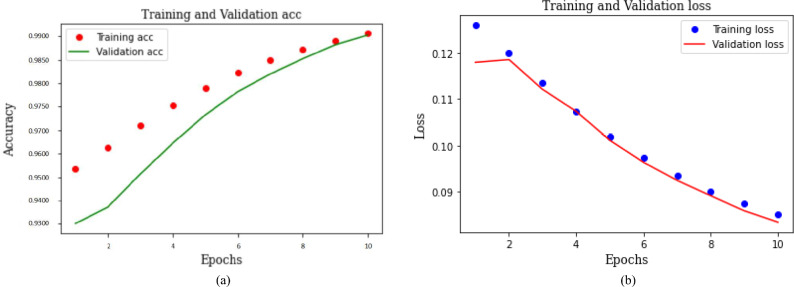
Training and validation performance of proposed CNN-GRU model for binary classification.

Figure 8 illustrates the training and validation performance for binary classification. Figure 8a represents accuracy curves while Fig. 8b depicts loss curves. The red dotted curve in Fig. 8a shows the training accuracy, and the green curve exhibits the validation accuracy. The model training is stopped after 10 epochs, as the accuracy begins to stabilize. It can be observed that the model accuracy for both training and validation consistently improves with each epoch. After 10 epochs, it reaches the maximum value of 0.99 for both training and validation results. Similarly, the training and validation loss of the model gradually decreases, reaching as low as 0.05. The incremental changes in accuracy and loss for both training and validation affirm that the proposed model avoids overfitting on the training dataset. Additionally, it demonstrates gradual learning, a characteristic further substantiated by the overall model performance during the testing phase..

**Fig. 9:**
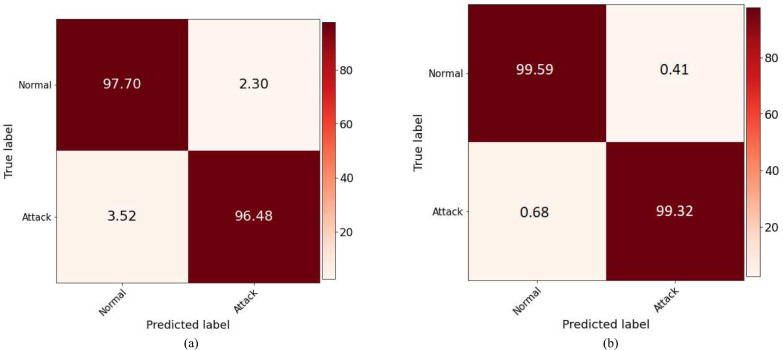
Confusion matrices for binary classification of normal versus attack scenario.

Figure 9 presents model performance for binary classification of normal versus attack scenarios. Confusion matrices in Fig. 9a and b show the detection rates before and after the application of the FW-SMOTE-based data augmentation technique, respectively. Figure 9a shows that the model correctly classifies 97.70% samples as belonging to the normal class and 96.48% as true attack samples. However, it misclassifies 3.52% of intrusion samples as normal and 2.30% of normal samples as an attack, indicating that, due to an imbalanced dataset, the model struggles to learn minority class samples effectively. The results for the FW-SMOTE oversampled dataset show an improvement, as depicted in Fig. 9b. It reduces the percentage of misclassified attack samples to 0.68% and normal samples to 0.41%. Consequently, the percentage of correct predictions for both normal and attack binary classification increases to 99.59% and 99.32% respectively.

Table 3 provides a performance comparison of given approaches for binary classification, distinguishing intrusion from normal samples. Our proposed model demonstrates exceptional performance, achieving the highest accuracy of 99.60% among all models. Additionally, the precision (99.17%), and F1-score (99.09%) of our proposed model also outperform other models, except for recall (99.02%). Comparatively, the recall metric for the CNN-LSTM approach slightly surpasses that of the proposed CNN-GRU with values of 99.15% and 99.02% respectively. Despite this, the proposed model exhibits significantly better precision, leading to a superior F1-score. The overall evaluation indicates that RNN performs the least effectively among the considered models. The improved performance of the CNN-LSTM hybrid model can be attributed to its ability to extract complex features that might be overlooked by individual models. This underscores the importance of hybrid architectures in enhancing the classification capabilities of intrusion detection systems.

**Table 3:** Comparative analysis of purposed (CNN-GRU) model with state-of-the-art deep learning techniques for binary attack classification.

Technique	Dataset	Accuracy (%)	F1-score (%)	Precision (%)	Recall (%)
CNN	IoTID20	97.08	97.15	97.16	97.14
RNN	IoTID20	96.34	96.28	96.72	95.87
LSTM	IoTID20	96.94	96.93	97.55	96.31
GRU	IoTID20	98.95	98.38	98.61	98.15
CNN-LSTM	IoTID20	98.01	98.79	98.40	99.15
Proposed (CNN-GRU)	IoTID20	**99.60**	99.09	**99.17**	99.02
	UNSW-NB15	99.14	**99.13**	99.13	**99.13**

Significant values are in bold.

To evaluate the effectiveness of the proposed model, we conducted training and testing using the UNSW-NB15 dataset to identify intrusion attacks. A comparative analysis between the IoTID20 and UNSW-NB15 datasets is presented in Table 3. The results demonstrate that the proposed model achieves a notable accuracy of 99.14% for attack detection on the USBW-NB15 dataset, showcasing its efficacy in the network domain. However, IoTID20 outperforms USBW-NB15 in both accuracy (99.60% > 99.14%) and precision (99.17% > 99.13%). Conversely, USBW-NB15 exhibits better performance in terms of recall (99.13% > 99.02%) and F1-score (99.13% > 99.09%). This indicate that the IoTID20 dataset produces a higher number of false negatives compared to the UNSW-NB15 dataset. Consequently, despite the higher precision metric on the IoTID20 dataset (99.17% > 99.13%), the F1-score is slightly better for UNSW-NB15 (99.13% > 99.09%). This highlights the competitive model performance in detecting network intrusions compared to IoT intrusions. Additionally, these results establish the model’s relevance and applicability in various domains.

The comparative analysis of the proposed model is conducted with existing studies in Table 4. Notably, the majority of studies on intrusion detection primarily focus on network datasets, with only a few addressing IoT domain datasets. The results show that our model outperforms existing studies, achieving the highest accuracy at 99.60%. In contrast, the accuracy is reported as 98.90% for NSL-KDD network dataset^62^ and 98.20% for the BoT-IoT dataset^63^. Although all the benchmark models are hybrid, the overall performance of our proposed model on IoTID20 stands best in the context of IoT intrusion detection. Hence, we recommend CNN-GRU model as a preferred choice for intrusion detection in IoT networks.

**Table 4:** Comparative analysis of purposed (CNN-GRU) model with existing studies for binary attack classification.

Authors	Model	Dataset	Dataset domain	Accuracy (%)	F1-score (%)	Precision (%)	Recall (%)
Liu et al.^62^	CNN-LSTM	NSL-KDD	Network	98.90	–	–	–
Alsudani et al.^49^	TLBO-ELM	NSL-KDD		98.21	–	–	–
Abdel-Basset et al.^40^	SS-DeepID	CIC-IDS2018		98.33	98.85	98.78	98.91
Ferrage et al.^63^	RNN-BPTT	BoT-IoT	IoT	98.20	–	–	–
Alkahtani et al.^38^	CNN-LSTM	IoTID20		98.0	98.80	98.40	**99.20**
Proposed	CNN-GRU	IoTID20		**99.60**	99.09	**99.17**	99.02
	CNN-GRU	UNSW-NB15	Network	99.14	**99.13**	99.13	99.13

Significant values are in bold.

### Multiclass attack type classification

**Fig. 10:**
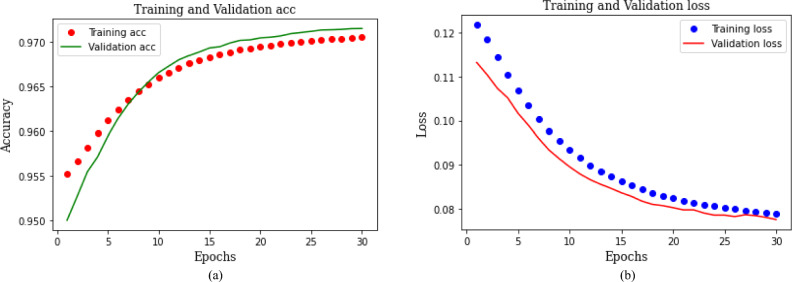
Training and validation performance of proposed CNN-GRU model for attack type classification.

The model performance for classifying the attack type is provided in Fig. 10. Figure 10a illustrates it for accuracy and Fig. 10b depicts it for loss. Both the training and validation curves exhibit the learning trajectory of the model. The accuracy of both the training and validation sets demonstrates a progressive enhancement as the number of epochs increases, while the loss steadily declines. Existing literature suggests that multiclass classification necessitates extensive training, particularly when dealing with large datasets. After thorough experimentation with various parameters, we have determined that our model achieves optimal performance when trained for 30 epochs. The hybrid CNN-GRU model achieves an accuracy of 0.97 for the categorical classification of attacks, with a model loss of 0.08.

**Fig. 11:**
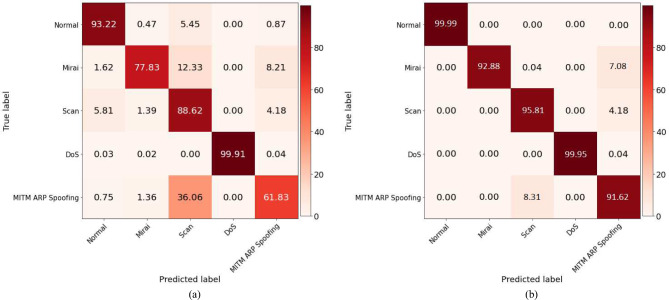
Confusion matrices for attack type classification.

Figure 11a and b demonstrate the classification performance for attack types. The validation set for intrusion type classification comprised 207,655 samples. Figure 11a shows that 6.79% normal class samples are misclassified as attack types, while 93.22% samples are correctly predicted. Similarly, for Mirai attack samples, 22.16% are mispredicted, and 77.83% samples are correctly classified. The misclassified samples for Scan, DoS, and MITM attacks are 11.38%, 0.09%, and 38.17%, respectively. The high misclassification percentage for attack types indicates the suboptimal performance of the model on the imbalanced dataset. However, the model exhibits significant improvement when applied to the FW-SMOTE oversampled dataset. Figure 11b shows that only 0.01% of normal class samples are misclassified, with 99.99% are correctly identified as normal samples. Similarly, our proposed approach enhances the detection rate for Mirai (92.88%), Scan (95.81%), DoS (99.95%), and MITMattack (91.62%) attack types. These results underscore the superior model performance on the balanced dataset compared to an imbalanced dataset, supporting the hypothesis that a balanced dataset leads to improved model training and enhances the detection rate for various attack types.

Similarly, Table 5 shows the comparative analysis for multiclass classification of different attack types. The CNN-GRU hybrid model achieved an accuracy of 98.15% in identifying the type of intrusion. In comparison, benchmark techniques such as CNN, RNN, LSTM, GRU and CNN-LSTM exhibited accuracies of 95.56%, 94.16%, 95.84%, 96.83% and 97.11% respectively. Therefore, our approach attained superior performance when compared to existing models for intrusion type classification. Consistent outcomes are observed across other evaluation metrics, where the proposed CNN-GRU model outperformed existing state-of-the-art models. Specifically, precision, recall and F1-score for the proposed CNN-GRU models are reported as 97.23%, 97.73% and 97.51%. In contrast, these metrics for CNN-LSTM are 96.89%, 96.83% and 96.86%. Although the proposed model performs superior, the CNN-LSTM model trails slightly in terms of precision compared to the CNN-GRU model.

**Table 5:** Comparative analysis of purposed (CNN-GRU) model with state-of-the-art deep learning techniques for attack type classification.

Technique	Dataset	Accuracy (%)	F1-score (%)	Precision (%)	Recall (%)
CNN	IoTID20	95.56	93.91	93.79	94.57
RNN	IoTID20	94.16	93.95	93.53	92.67
LSTM	IoTID20	95.84	93.53	95.42	92.03
GRU	IoTID20	96.83	95.16	95.59	94.69
CNN-LSTM	IoTID20	97.11	96.86	96.89	96.83
Proposed (CNN-GRU)	IoTID20	**98.15**	**97.51**	97.23	**97.73**
	UNSW-NB15	97.56	97.49	**97.61**	97.57

Significant values are in bold.

The model validation results for attack type classification on the UNSW-NB15 are presented in Table 5. The findings highlight the competitive performance of the hybrid CNN-GRU model on the UNSW-NB15 dataset. While the accuracy for the UNSW-NB15 dataset is slightly lower compared to the IoTID20 dataset, it still falls within the range of satisfactory performance. The precision is higher for UNSW-NB15 (97.61% > 97.23%), but due to a lower recall value (97.73% < 97.57%), the F1-score lags by a narrow margin (97.49% < 97.51%). Despite these marginal differences, the overall competitive performance on the UNSW-NB15 dataset justifies model applicability across different domains.

### Multiclass attack subtype classification

**Fig. 12:**
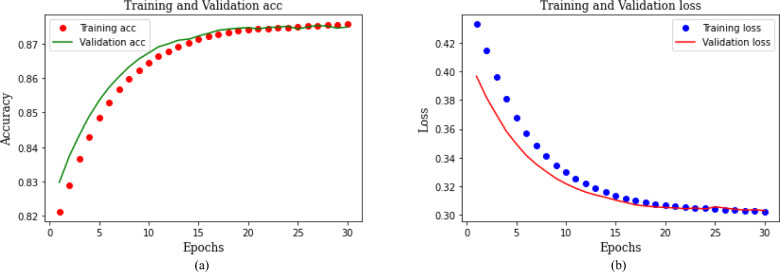
Training and validation performance of proposed CNN-GRU model for attack subtype classification.

Likewise, the model performance for attack subtype classification is shown in Fig. 12. Figure 12a depicts the accuracy, while Fig. 12b demonstrates loss for the attack subtype. The proposed model demonstrates an accuracy of 0.88 for both training and validation cases at 30 epochs. The loss of the CNN-GRU model is computed at each epoch and the plot shows a loss of 0.30 after 30 epochs of training. The convergence of training and validation curves towards the end of the plot indicates that the model is not overfitting to the training data but rather learning effectively.

**Fig. 13:**
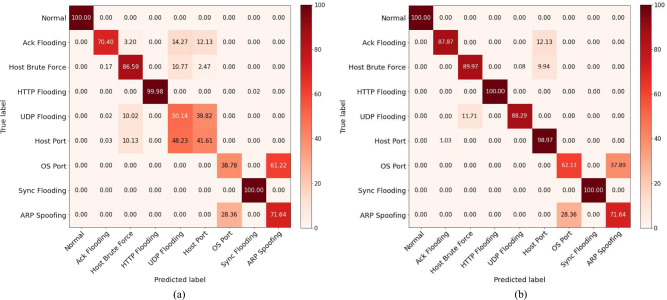
Confusion matrices for attack subtype classification.

For the subcategorical classification of attack types, Fig. 13 depicts the model performance before and after applying the FW-SMOTE oversampling technique. In Fig. 13a attack subtype detection rates are depicted for an imbalanced dataset while Fig. 13b provides it for the balanced dataset. The results show that attack subtypes such as ACK Flooding, UDP Flooding, Host Port, OS Port, and ARP Spoofing misclassify a significant percentage of validation samples for the imbalanced dataset. When analysed after applying the FW-SMOTE oversampling technique, all subtype classification results exhibit significant improvement, except for the APR Spoofing subtype. The detection rate for ARP Spoofing remained stable (71.64%) for both imbalanced and balanced datasets. This affirms that a balanced dataset improves model performance across all three cases analysed for intrusion detection in IoT networks. The utilization of an imbalanced dataset degrades model performance due to the biased performance of the model against minority class samples.

The overall model performance is presented in Table 6. The results reveal that the hybrid CNN-GRU model achieves the highest overall accuracy at 86.76% for the classification of attack subtypes. When compared to alternative deep learning-based methods, our proposed model consistently outperforms them across all performance evaluation metrics. Specifically, the accuracy values for CNN, RNN, LSTM, GRU, and CNN-LSTM are 80.18%, 78.18%, 82.65%, 82.83%, and 82.96%, respectively. Similarly, the F1-score, which reflects the cumulative performance in terms of both precision and recall metrics, also demonstrates the superior performance of the CNN-GRU model. The hybrid model achieves an F1-score of 85.55%, surpassing the CNN-LSTM score of 84.14% by a significant margin. The remaining benchmark approaches exhibit even worse performance compared to the hybrid models.

**Table 6:** Comparative analysis of proposed (CNN-GRU) model with state-of-the-art deep learning techniques for attack subtype classification.

Technique	Dataset	Accuracy (%)	F1-score (%)	Precision (%)	Recall (%)
CNN	IoTID20	80.18	81.20	80.40	81.89
RNN	IoTID20	78.18	78.95	78.40	79.89
LSTM	IoTID20	82.65	80.23	80.43	79.97
GRU	IoTID20	82.83	83.91	83.59	84.27
CNN-LSTM	IoTID20	82.96	84.14	85.25	83.54
Proposed (CNN-GRU)	IoTID20	**86.76**	**85.55**	**87.28**	**85.25**

Significant values are in bold.

### Detection time analysis

The proposed model is also evaluated for detection time assessment on both the datasets IoTID20 and UNSW-NB15. Table 7 presents the average detection time of the model in milliseconds (ms). The results show that the hybrid CNN-GRU model demonstrates better performance on the network domain dataset UNSW-NB15 than the IoTID20 dataset. The average intrusion detection time on the UNSW-NB15 dataset is 88 ms, whereas it is 94 ms on the IoTID20 dataset. Similarly, for attack type, the proposed model excels on the UNSW-NB15 dataset by attaining 93 ms detection time whereas the IoTID20 dataset exhibits suboptimal performance on the proposed model, trailing with a margin of 1.5 ms.

The average detection results for subtypes are missing for the UNSW-NB15 dataset due to the unavailability of labelled data for subtypes of attacks. However, CNN-LSTM outperforms the proposed CNN-GRU model by a margin of 3 ms. UNSW-NB15 has 37 features while IoTID20 has 51 features which increases the computational complexity of the model on the IoTID20 dataset. The intrusion detection time on the IoTID20 dataset highlights the trade-off between detection accuracy and detection time. The proposed hybrid CNN-GRU model achieves significantly superior detection accuracy with a marginally higher detection time. Conversely, CNN-LSTM achieves a better detection time with lower detection accuracy of the attacks.

**Table 7:** Average intrusion detection time (ms) comparison of proposed (CNN-GRU) model with state-of-the-art deep learning techniques.

Technique	Dataset	Binary (ms)	Attack type (ms)	Attack subtype (ms)
CNN	IoTID20	98	113.7	178.1
RNN	IoTID20	153	168	174
LSTM	IoTID20	154	169.1	174.1
GRU	IoTID20	122	97.7	103.4
CNN-LSTM	IoTID20	92	95	**95.1**
Proposed (CNN-GRU)	IoTID20	94	96.5	98
	UNSW-NB15	**88**	**93**	–

Significant values are in bold.

It is evident from Table 7 that the computational complexity of the proposed model is less as compared to the state-of-the-art models. The complexity of CNN operations is influenced by the size of input data, number of filters, kernel sizes, and depth of the network which we have optimized in the proposed model. Following the CNN stage, GRUs are employed to capture temporal dependencies in sequential data, adding another layer of computational demand due to recurrent connections and sequential processing. Efficient implementation and optimization of these components helped us in achieving real-time performance in intrusion detection while managing computational resources effectively.

## Discussion

The main limitation of most existing ML/DL models lies in their tendency to focus primarily on network intrusion detection attacks, utilizing datasets and methodologies tailored specifically for countering such attacks. IoT intrusion detection systems, on the other hand, are designed to align with the unique characteristics of IoT networks. In most of the IoT intrusion detection research, the evaluation typically focuses on binary or multiclass classification, neglecting consideration for diverse intrusion types. The stability of ML-based approaches is constrained by the robustness of the employed handcrafted features. In contrast, DL approaches suffer from suboptimal performance rates due to the presence of redundant and irrelevant features. Despite the numerous research studies proposing effective DL-based approaches, none of the models put forth to date simultaneously identify the attack, attack type, and subtype. Furthermore, imbalanced classes significantly impact the models, resulting in increased misclassification rates.

Therefore, this study identifies the limitations of existing IoT-based intrusion detection systems and proposes a solution to overcome these limitations. The intricacy and variety of intrusion patterns in IoT networks often demand models with greater capacity to capture complex patterns in the data. The decision to use the proposed model for intrusion detection stemmed from the necessity for enhanced capacity, accuracy, and the capability to effectively detect and classify various anomaly patterns within the IoT network. We initially explored and implemented a shallow network for intrusion detection, but it did not yield the anticipated result. Consequently, considering the aforementioned factors, we opted for implementing a deeper model.

We propose a hybrid CNN-GRU deep learning-based approach to simultaneously identify IoT attacks, their types, and the subtypes, contributing to an effective IoT Intrusion Detection System (IDS). In our hybrid model, CNN is employed to extract higher-order features and local features from IoT activities, while GRU delves into analyzing contextual information and the relational aspects of feature patterns. The PSO is employed for selecting the significant features from the input feature vector for improving model generalization. PSO enhances CNN-GRU based intrusion detection systems by optimizing hyperparameters, such as learning rates and layer sizes, to improve model performance and generalization across different intrusion scenarios and datasets. PSO also aids in feature selection, focusing on the most discriminative inputs to mitigate overfitting and enhance the model’s ability to detect relevant intrusion patterns. The model is tested using the recently proposed IoTID20 dataset, widely adopted in the research community for modelling IoT intrusion detection systems. An FW-SMOTE-based data augmentation is implemented to address the class imbalance issue in the IoTID20 dataset.

The effectiveness of the proposed solution is validated through comparisons with the existing state-of-the-art models. Three separate simulations are conducted and evaluated in three distinct scenarios. The outcomes of the simulations imply that the proposed scheme enhances the security of IoT applications by accurately detecting attacks and identifying their types and subtypes. The model excels in attack detection for both IoT and network intrusion detection domains, and it outperforms state-of-the-art approaches in attack type and subtype classification. Implied from the results, we are hopeful regarding the model’s applicability in diverse domains. In IoT applications, where data streams can be vast and varied, CNNs excel at spatial feature extraction from sensor data or image inputs, while GRUs effectively model temporal dependencies in sequential data such as sensor readings or network packets. This dual capability allows for robust intrusion detection across different types of IoT devices and network environments. However, adapting the CNN-GRU model to different network environments necessitates careful tuning and possibly retraining on domain-specific datasets to maintain detection accuracy amidst evolving intrusion tactics and network behaviors.

The PSO involves iterative optimization over a potentially large parameter space, which can be time-consuming and resource-intensive, especially for complex models and large datasets typical in intrusion detection. This complexity may limit real-time deployment capabilities, particularly on resource-constrained IoT devices or in high-speed network environments where rapid decision-making is crucial. Scalability can also be an issue, as PSO’s effectiveness may diminish with increasing dataset size or model complexity, necessitating careful tuning and possibly compromising detection accuracy in diverse or evolving intrusion scenarios.

It has been observed that most of the misclassifications made by the model are the sequences that have similar patterns and the model has confused them with each other. Another reason is that since GRUs are used in conjunction with CNNs, the model misclassifies sequences where the temporal dependencies are not effectively captured. This could happen if the CNN features do not adequately represent the sequence context for the GRU to learn from the data.

## Conclusion

IoT networks may be vulnerable to threats due to weak protective measures and the ubiquitous nature of complex cyber-attacks. It is essential to identify such complex cyber-attacks to build sustainable IoT networks. Intrusion detection systems (IDSs) provide effective security measures against various types of attacks on IoT networks. This work has proposed a statistical learning-based intrusion detection model called hybrid CNN-GRU. The proposed approach consists of hybrid feature learning approaches such as CNN and GRU to detect attacks launched from different kinds of smart IoT devices in IoT networks. It is accomplished by collecting and classifying the IoT network traffic’s normal behaviour. The proposed method is validated using the IoTID20 benchmark dataset, specifically designed for the IoT environment, and can classify different types and subtypes of attack events. Additionally, to validate the cross-domain effectiveness, the proposed approach is also tested on the network domain dataset UNSW-NB15. The study results prove our hypothesis that the proposed model has a higher detection accuracy when compared to current IoT-enabled IDSs. The achieved accuracy for the IoTID20 dataset is 99.60% and is 99.14% for the UNSW-NB15 dataset. Similarly, the detection accuracy for attack type classification is 98.15% for the IoTID20 dataset and 97.56% for the UNSW-NB15 dataset. The results for attack subtype classification are only available from the IoTID20 dataset with a value of 86.76%. There exists a trade-off when proposed model is analysed for average intrusion detection time on IoTID20 and UNSW-NB15 datasets. Due to the bigger and heterogeneous feature space of the IoTID20 dataset, it achieved better accuracy at the cost of higher detection time in contrary to the UNSW-NB15 dataset which displayed better detection time with low detection accuracy.

Although the proposed hybrid model in this work brings a significant contribution to the IoT intrusion detection domain and extends its scope to classify types and subtypes of attack. Recently proposed transformer architecture has the potential to improve existing methodologies by employing attention mechanisms to extract localised features. By utilising transformer architecture to identify long-range dependencies and using attention techniques to rank pertinent features in system logs or network traffic, an intrusion detection system based on CNN-GRU can achieve notable improvements. The transformer can effectively adapt to a variety of incursion patterns by eliminating the requirement for manually constructed features by learning hierarchical representations of data. Through the integration of CNNs for extracting geographic features, GRUs for modelling temporal sequences, and transformers for comprehending global context, the system can attain exceptional results in real-time intrusion detection, hence augmenting cybersecurity resilience.

In future, we can extend this work to apply a transfer learning approach and use domain-specific pre-trained models for intrusion detection. These pre-trained models can be acquired from HuggingFace and Keras.io platforms. Transfer learning leverages already extracted features to improve detection results and resolves the small and imbalanced dataset limitations. Another avenue for improvement is to reduce intrusion detection time with deep neural networks (DNN). DNNs typically entail more parameters which increases the computation time of the model. The potential research guideline is to manage a trade-off between achieving high detection accuracy with a low computation cost. This will ascertain the feasibility of applying intrusion detection models in real-time scenarios. In situations where there is scarce labelled intrusion data, applying knowledge from pre-trained models-such as those trained on system logs or general network traffic-to intrusion detection tasks can expedite training and improve detection accuracy. It is imperative to optimise DNN structures and methods for streaming data processing in real time. Effective methods to reduce detection latency include model reduction, quantization, and parallelization with GPUs or TPUs. When combined, these techniques quicken the detection process, allowing for quick reactions to emerging threats and enhancing overall cybersecurity resilience.

## Data Availability

All data generated or analyzed during this study are available from the corresponding author upon reasonable request.
